# Large-Area Binary Blazed Grating Coupler between Nanophotonic Waveguide and LED

**DOI:** 10.1155/2014/586517

**Published:** 2014-07-13

**Authors:** Hongqiang Li, Wenqian Zhou, Meiling Zhang, Yu Liu, Cheng Zhang, Enbang Li, Changyun Miao, Chunxiao Tang

**Affiliations:** ^1^School of Electronics and Information Engineering, Tianjin Polytechnic University, No. 399, Binshuixi Road, Xiqing District, Tianjin 300387, China; ^2^School of Physics, Faculty of Engineering and Information Sciences, University of Wollongong, Wollongong, NSW 2522, Australia

## Abstract

A large-area binary blazed grating coupler for the arrayed waveguide grating (AWG) demodulation integrated microsystem on silicon-on-insulator (SOI) was designed for the first time. Through the coupler, light can be coupled into the SOI waveguide from the InP-based C-band LED for the AWG demodulation integrated microsystem to function. Both the length and width of the grating coupler are 360 *μ*m, as large as the InP-based C-band LED light emitting area in the system. The coupler was designed and optimized based on the finite difference time domain method. When the incident angle of the light source is 0°, the coupling efficiency of the binary blazed grating is 40.92%, and the 3 dB bandwidth is 72 nm at a wavelength of 1550 nm.

## 1. Introduction

**Figure 1:**
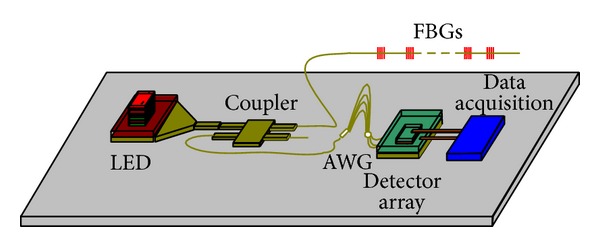
The proposed AWG demodulation integration microsystem.

Fiber Bragg grating (FBG) sensors are widely known to have achieved great progress. These sensors are used to measure strain, temperature, pressure, and other physical quantities that can be converted into strain or temperature [1]. The fiber grating demodulation system based on arrayed waveguide grating (AWG) is a new potential method that can be used for fiber grating demodulation, which is smaller and cheaper than the conventional fiber grating demodulation system [2]. The current work aims to study the integration of AWG demodulation system to achieve an AWG demodulation integrated microsystem. The proposed AWG demodulation integrated microsystem is shown in Figure 1. Light source is very important for the integrated microsystem, and silicon-on-insulator- (SOI-) LEDs are difficult to achieve under the current process [3]. In this work, the InP-based C-band LED and SOI waveguide are bonded by benzocyclobutene (BCB) using heterogeneous integration.

Grating couplers are widely used to couple light between nanophotonic waveguides and free space optical components. The coupling efficiency of a conventional fiber-to-chip grating coupler, however, is at a maximum of 40% to 60% for a standard SOI with an incident angle of 0° [4–7]. In recent years, SOI fiber-to-chip grating couplers have been widely reported [8, 9]. Many methods have been proposed to improve the coupling efficiency of the grating coupler [10–13], and blazed grating coupler is widely studied [14–17]. Binary blazed grating coupler has higher coupling efficiency with vertical incident light and needs not additional complexity in the manufacture process compared to the uniform grating coupler. Grating coupler used for the integration of VCSELs and photodetectors onto SOI waveguide circuits was relatively little reported [18, 19]. Most couplers are used between the waveguide and the optical fiber, rarely between the waveguide and the LEDs. In addition, for optoelectronic heterogeneous integrated systems such as the InP-based C-band LED in the FBG demodulation system, the size of common fiber-to-chip grating couplers is usually not a large enough size.

In this work, we first designed a large-area binary blazed grating coupler with a high coupling efficiency and a wide bandwidth. Both the length and width of the coupler are 360 *μ*m, as large as the InP-based C-band LED light emitting area in the AWG demodulation integration microsystem. The coupler was simulated and optimized based on the finite difference time domain (FDTD) method.

## 2. Design and Simulation

**Figure 2:**
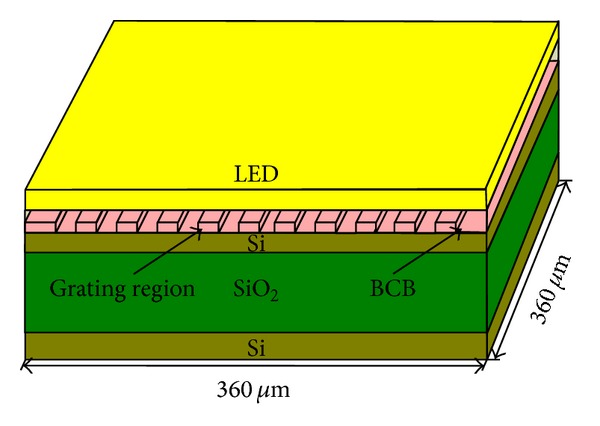
The proposed binary blazed grating structure.

SOI, the selected material for this work, is a well-known platform for microelectronics and optoelectronics. As a material used in waveguide devices, SOI displays superiority in the following aspects: compatibility with silicon processing, convenience for electronic integration and photonic integration, waveguide characteristics, fast operation in optical circuit, and radio protection. SOI can be used in optical device interconnection and can be applied in military devices. Extremely small devices can be fabricated on SOI substrates because of the ultrahigh refractive index between Si and SiO_2_. The grating couplers were designed for an FBG demodulation system that was fabricated on a standard SOI wafer with a top silicon thickness (*d*_si_) of 0.22 *μ*m and a buried oxide thickness (*d*_sio_2__) of 2 *μ*m. The refractive indices of the silicon layer and the buried oxide layer are 3.46 and 1.45, respectively. In addition, the refractive indices and thickness of BCB in this structure are 1.544 and *d*_BCB_, respectively. The proposed binary blazed grating structure is shown in Figure 2.

**Figure 3:**
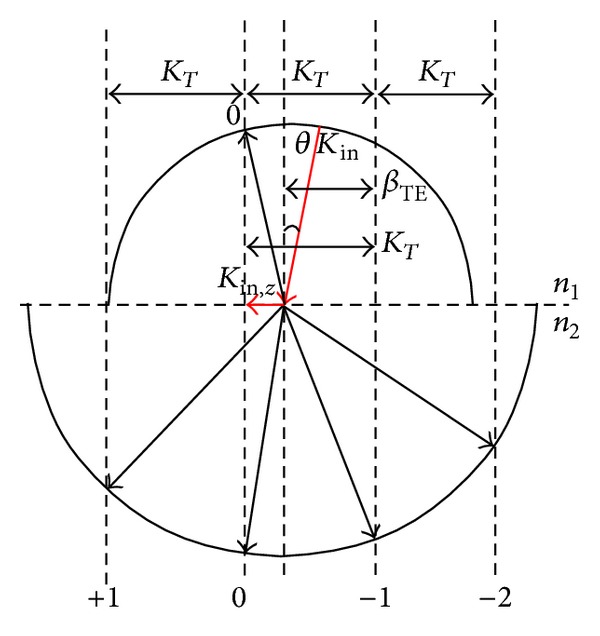
Wave-vector diagram for the grating coupler.

Grating coupling is realized by the diffraction of light. According to the Bragg condition, the diffraction order and the propagation direction can be analyzed. The wave vector diagram for the Bragg condition is shown in Figure 3. The Bragg condition introduces the concept of grating vector and describes the relationship of the vector between the incident wave and the respective diffraction order. The mode of the grating vector along the longitudinal direction of the grating is

(1) $$\begin{array}{c} \left|\vec{K_{T}}\right|=\frac{2\pi }{T}, \end{array}$$

where *λ* is the wavelength of the light and *T* is the grating period.

For the distribution of the grating structure in the interface of two materials, the refractive index of the two materials is, respectively, *n*_1_ and *n*_2_. In this case, the wave vector of *m*-order diffracted light is as follows:

(2) $$\begin{array}{c} \vec{K_{\text{in}}}+m\vec{K_{T}}=\beta ,\;\left(m=0,\pm 1,\ldots \right). \end{array}$$

For the grating structure on the dielectric waveguide, the propagation constant of the waveguide mode is

(3) $$\begin{array}{c} \beta =k_{0}N_{\text{eff}}=\frac{2\pi }{\lambda }N_{\text{eff}}, \end{array}$$

where *N*_eff_ is the effective refractive index of the grating region, which can be obtained from the mode dispersion equations of slab waveguide

(4) (n22−Neff2)1/22πλH=mπ+tan−1(Neff2−n12n22−Neff2)1/2+tan−1(Neff2−n32n22−Neff2)1/2.

Using this theory, the formula for grating period commonly used for micro-nano-waveguide grating coupler can be obtained:

(5) $$\begin{array}{c} T=\frac{m\lambda }{N_{\text{eff}}\mp n_{1}\cdot \sin\theta },\;\left(m=0,\pm 1,\ldots \right). \end{array}$$

In (5), *θ* is the angle of incidence; the positive sign is for the optically coupled direction of positive, and the negative sign is for the optically coupled direction of negative. For the conventional grating coupler, the grating period can be estimated via the Bragg condition, whereas the other parameters of the grating, such as the grating length, the shape, width, and depth of the grating grooves, are not mentioned. Coupling efficiency, another vital parameter index of grating couplers, cannot be calculated or estimated by the formula.

**Figure 4:**
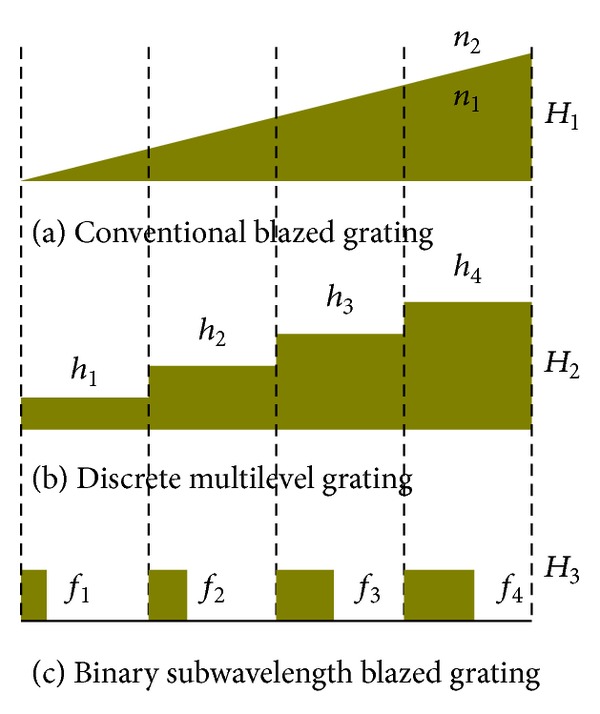
Blazed grating coupler discretization process. (a) Common blazed grating. (b) Discrete multilevel grating. (c) Binary grating.

Every period of the binary grating is equally divided into *M* subperiods with the width of Λ = *T*/*M*. The fill factor of each subperiod *f*_*i*_  (*i* = 1,2, 3,…, *M*) is defined as the ratio of pillar width to grating subperiod. The width of each pillar can be controlled to obtain the desired refractive index distribution. The basic design procedure and discrete processing are shown in Figure 4.

As shown in Figure 4, *H*_1_ is the height of the common blazed grating, *h*_*i*_  (*i* = 1,2, 3,4) is the height of each discrete multilevel grating, *H* denotes the height of the binary subwavelength blazed grating, and *n*_eff(*i*)_^TE^ is the effective refractive indices of the binary grating:

(6) $$\begin{array}{c} h_{i}=\frac{1}{2}\left[\frac{H_{1}}{M}\cdot i+\frac{H_{1}}{M}\left(i-1\right)\right]=\frac{\left(2i-1\right)H_{1}}{2M} \end{array}$$

(7) $$\begin{array}{c} n_{\text{eff}\left(i\right)}^{\text{TE}}=\frac{h_{i}}{H}n_{2}+\frac{H-h_{i}}{H}n_{1}. \end{array}$$

Based on form-birefringence theory, the effective index of TE mode in the grating is determined by

(8) $$\begin{array}{c} n_{\text{eff}\left(i\right)}^{\text{TE}}=\sqrt{f_{i}n_{2}^{2}+\left(1-f_{i}\right)n_{1}^{2}}. \end{array}$$

Equations (6) to (8) show that fill factors can be computed as follows:

(9) fi=[((2i−1)/2M)(H1/H)(n2−n1)+n1]2−n12n22−n12,(i=1,2,3,…,M),

where *H*_1_ is the height of the common blazed grating, *H* denotes the height of the binary subwavelength blazed grating, and *n*_2_ and *n*_1_ are the refractive index of Si and BCB, respectively.

According to (5), assuming that light shines perpendicularly on the grating and the diffraction order *m* = 1, on the basis of this original value 560 nm, *T* is further optimized by scanning from 550 nm to 575 nm using the FDTD software to meet the optimal value 571 nm. In our structure, considering coupling efficiency and fabrication constraint, we set *M* = 2 and *H* = 0.07 *μ*m, respectively. According to (9), in order to ensure the fill factor *f*_*i*_ being less than 1, *H*_1_/*H* should not be greater than 2 *M*/(2 *M* − 1); that is, *H*_1_/*H* should not be greater than 4/3 when *M* = 2; we set *H*_1_/*H* = 1.15. Fill factors can be given by the following equation when *M* = 2:

(10) fi=[((2i−1)/4)(H1/H)(3.46−1.544)+1.544]2−1.54423.462−1.5442,(i=1,2).

**Figure 5:**
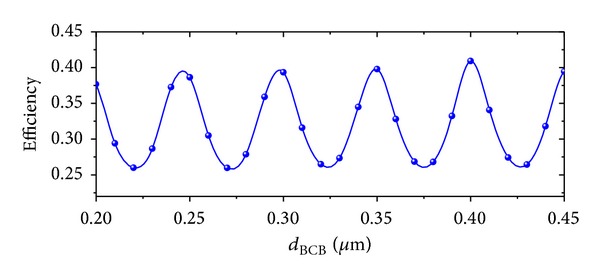
Coupling efficiency with *d*_BCB_.

**Figure 6:**
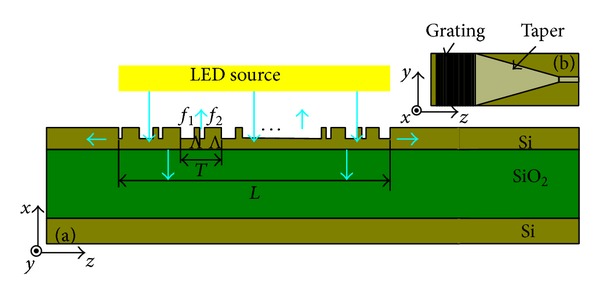
Schematic of the proposed binary blazed grating coupler: (a) top view and (b) side view.

In the two-dimensional FDTD simulation, the structure of InP-based C-band LED comprises a 1 *μ*m thick InP substrate, a 0.8 *μ*m thick n-InP buffer layer, a quantum well (QW) active layer, a 0.2 *μ*m thick p-InP limiting layer, and a 0.15 *μ*m thick p-InGaAsP top layer. A point dipole with the orientation of *z*-axes was located in the middle of the active layer and was used as the radiating source of the LED [20], primarily emitting radiation with a transverse electric (TE) polarization [21]. The refractive indexes of InP and InGaAsP were set as 3.29 and 3.64, respectively. The boundary conditions were set to the perfectly matched layer (PML). To achieve high coupling efficiency to the output waveguide, *d*_BCB_ is optimized by the FDTD software simulation. *d*_BCB_ is changed from 200 nm to 400 nm, and the coupling efficiency with *d*_BCB_ is shown in Figure 5. When *d*_BCB_ = 0.4 *μ*m, the coupling efficiency meets the maximum. Schematic of the proposed binary blazed grating coupler is shown in Figure 6. The output waveguide of the grating coupler will be connected to a taper acting as the mode converter to convert the light from multimode to single mode. The relative parameters of the binary blazed grating coupler are shown in Table 1.

**Figure 7:**
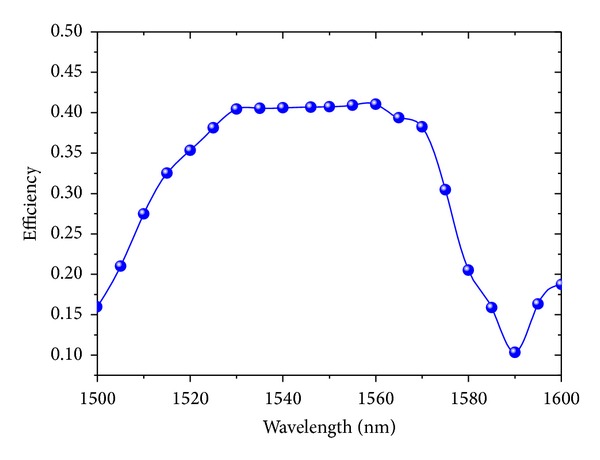
Wavelength and coupling efficiency at the incident angle of 0°.

**Figure 8:**
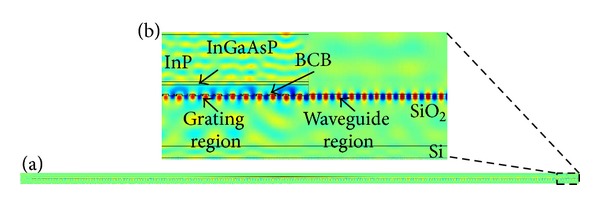
Optical field of the large-area binary blazed grating coupler bonded with the LED. (a) The optical field of the overall structure. (b) The optical field of the structure's output terminal.

The structure is simulated with an incident angle of 0°. The relationship between the coupling efficiency and the incident light wavelength is shown in Figure 7. The coupling efficiency has a maximum of 41.04% at a wavelength of 1560 nm. For the center with 1550 nm wavelength, the coupling efficiency is 40.92% with a 3 dB bandwidth of 72 nm, which overcomes the problem that the coupling bandwidth becomes narrower as the length of grating coupler increases, and on this occasion, the losses in the reflection, transmission to the SiO_2_ layer, and the coupling to the opposite direction are 45.30%, 6.27%, and 3.16%, respectively, with the losses of 4.35% in the free area. The optical field of the large-area binary blazed grating coupler bonded with the LED is shown in Figure 8. Obviously, a large part of light converges to the output terminal into the output waveguide with the reflection back into LED and transmission into the SiO_2_ layer.

## 3. Analysis and Discussion

According to the Bragg condition, the incident angle *θ* of the grating coupler has considerable influence on optical coupling. To facilitate system integration, most studies aim to reach *θ* = 0° with a high coupling efficiency and a wide bandwidth. However, for a subwavelength grating coupler, the incident angle is sensitive to the coupling efficiency. To avoid the negative effects of incident angle error on the coupling efficiency, the grating coupler must have better incident angle tolerance.

**Figure 9:**
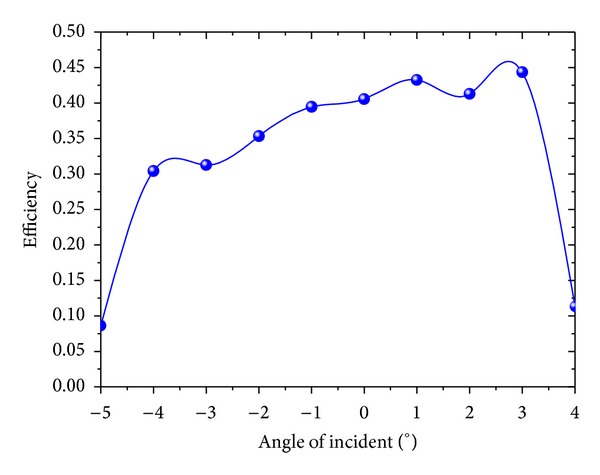
Light incident angle and coupling efficiency.

Under the condition of a different incident angle, the large-area binary blazed grating was simulated at a 1550 nm wave. The result obtained by 10 simulations is shown in Figure 9. When the incident angle is −1°, the coupling efficiency is 39.46%, and the coupling efficiency is 41.28% with an incident angle of 2°. The angle of incidence ranged from −1° to 2°, and the coupling efficiency was maintained at 39.46% or higher. That is to say, the grating coupler angle tolerance is 3°. For the current packaging process, the angle tolerance of 3° is enough. Though unnecessary, under the condition of vertical incidence, the incident angle can be set at 1° to achieve a higher coupling efficiency.

Of the elements in the blazed grating coupler, grating etching depth has great influence on the coupling efficiency. In the present semiconductor process, however, etching depth error relatively exists. To be able to better realize the optical coupling, subwavelength grating coupler etching tolerance should be considered. Higher etching tolerance will result in lower grating coupler manufacturing requirement for the process, and the cost will be cheaper.

**Figure 10:**
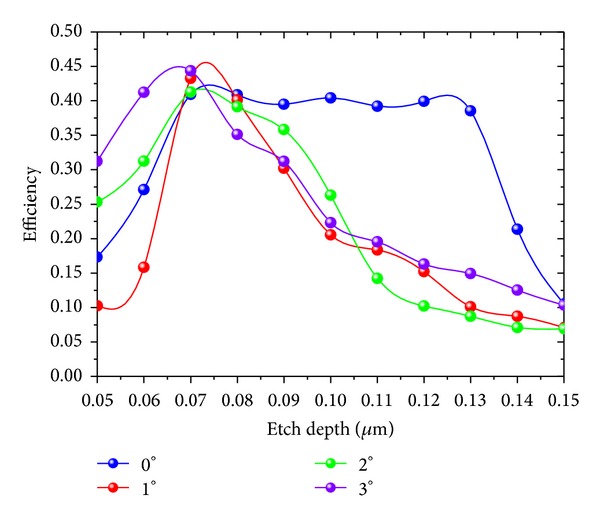
Coupling efficiency at different etch depths and incident angles.

Under the different incident angles, structures with different etching depths are simulated at a 1550 nm wave. The result obtained for four incident angle simulations is shown in Figure 10. Incident angle tolerance and etching depth tolerance have a mutual influence on the coupling efficiency. When the incident angle is 0°, and etching depth is increased from 0.07 *μ*m to 0.13 *μ*m, the coupling efficiency is 39% or higher, and the change is less than 2%. That is to say, when the incident angle is 0°, the grating structure etching tolerance is 0.06 *μ*m. This result greatly reduces the production process requirements. When the incident angle is 1°, the coupling efficiency is greater than 39% only under the condition that the etch depth is between 0.06 and 0.07 *μ*m. Similarly, the incident angle tolerance is small when the incident angle is 2°. When the incident angle is 3°, the etching tolerance is smaller than that at the incident angle of 0°; its coupling efficiency can be as high as 44.36%. Thus, when the etching precision is high, an incident angle of 3° can be chosen to improve the coupling efficiency. When the etching precision is at a general level, an incident angle of 0° can be set to guarantee the stability of the coupling.

## 4. Conclusion

In this work, a large-area binary blazed grating coupler between the InP-based C-band LED and SOI waveguide was designed. Both the length and width of the grating coupler are 360 *μ*m. These processes were done using the EastFDTD software of Dongjun Technology Co., Ltd. Each FDTD simulation model required 19 hours to complete. Finally, all the parameters of the grating coupler were obtained. The coupling efficiency can be as high as 40.92% with a 3 dB bandwidth of 72 nm at a wavelength of 1550 nm on the assumption of large size. In addition, the incident angle tolerance and etching depth tolerance are 3° and 60 nm, respectively.

## Figures and Tables

**Table 1:** Parameters of the binary blazed grating coupler.

Parameter	*f*_1_	*f*_2_	*T* (*µ*m)	*Λ* (*µ*m)	*L* (*µ*m)
Value	0.21	0.82	0.571	0.2855	360
